# Supplementary material for: Krill faecal pellets drive hidden pulses of particulate organic carbon in the marginal ice zone
Source: Nat Commun. 2019 Feb 21;10:889. doi: 10.1038/s41467-019-08847-1 (PMC6385259; doi:10.1038/s41467-019-08847-1)
Supplement: Supplementary file 1 — Supplementary Information [file 41467_2019_8847_MOESM1_ESM.docx]

**Krill faecal pellets drive hidden pulses of particulate organic carbon in the marginal ice zone**

A. Belcher et al.

**Supplementary Information**


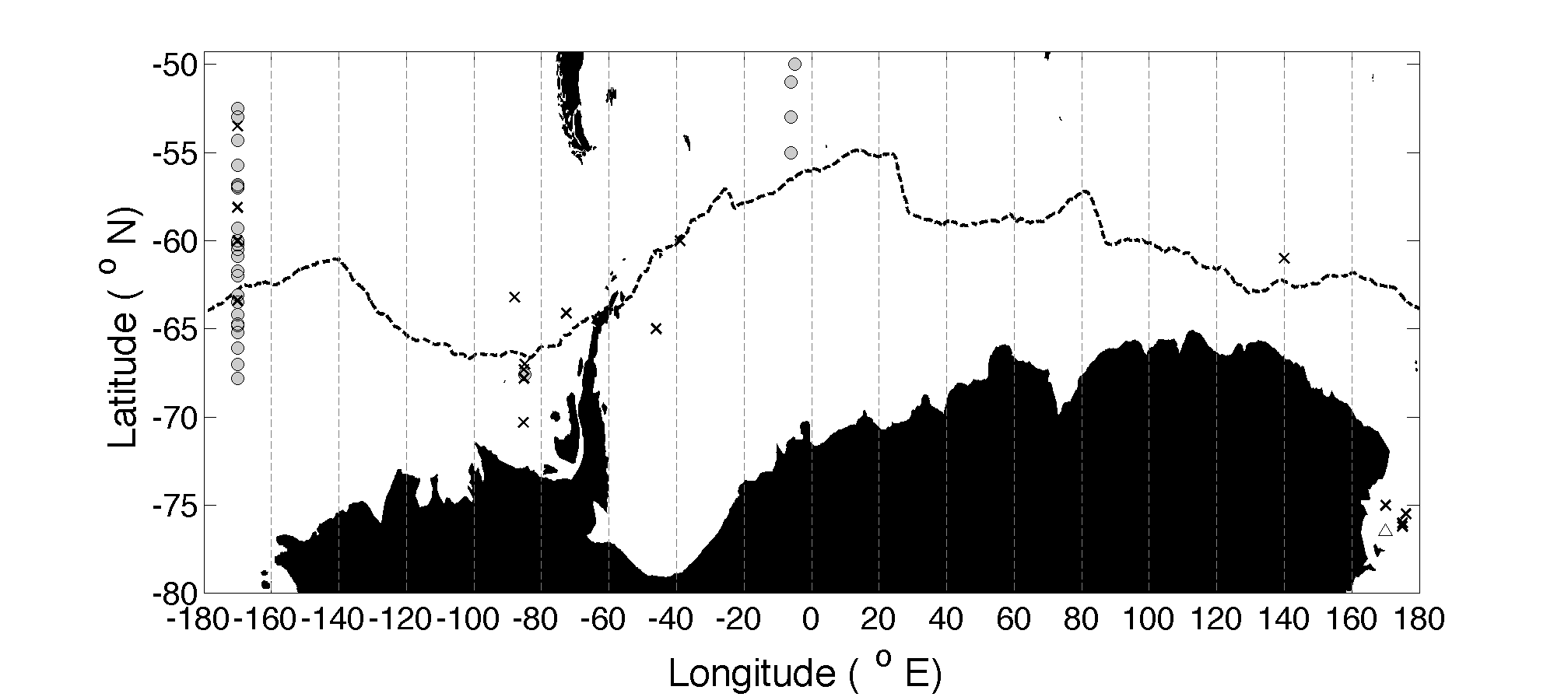


**Supplementary Figure 1: Locations of data used to derive algorithms for export production**. Positions of data from Henson^1^ (grey circles), Dunne^2^ (black crosses) and Laws^3^ (white triangle) are shown with reference to the maximum ice extent (black dashed line, 15% sea ice concentration during period October 1^st^-13^th^ 1994-2014).

**
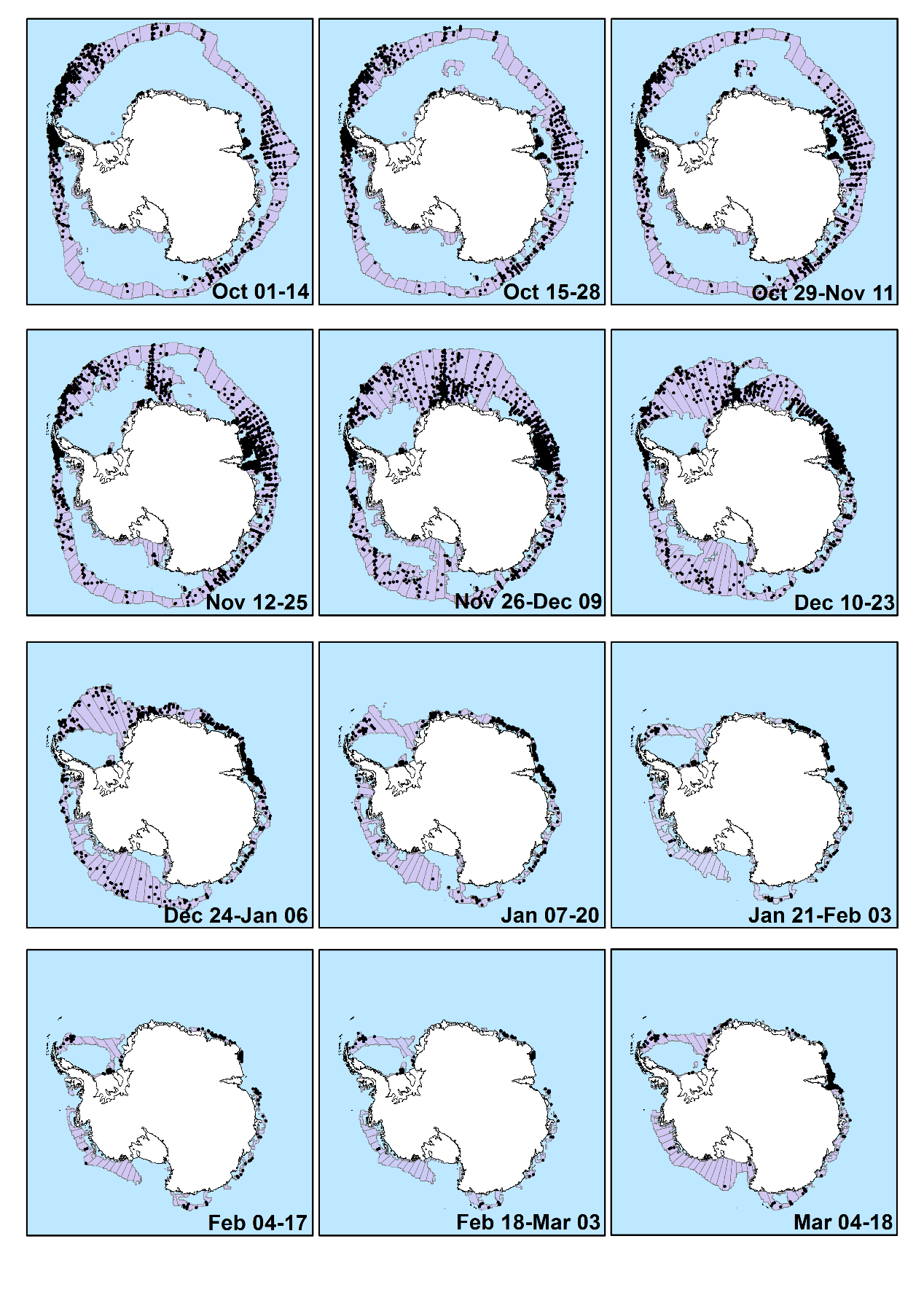
**

**Supplementary Figure 2: Location of krill density estimates from KRILLBASE (black dots) (projected to each fortnightly period) within the marginal ice zone (grey), Antarctica.** The marginal ice zone, from fortnightly sea ice concentration data (15-80% ice cover), is divided into 5 **°** zonal cells. The Antarctic coastline was obtained from the SCAR Antarctic Digital Database.

**Supplementary Table 1: Measurements of krill faecal pellet (FP) flux in the Southern Ocean.**

| **Source** | **Region** | **Depth (m)** | **Season** | **Krill FP flux**  **(mg C m^-2^ d^-1^, or *nFP m^-2^ d^-1^*) ^a^** | **Notes** |
| --- | --- | --- | --- | --- | --- |
| Belcher et al. (2017)^4^ | South Orkneys * | 64 | December | 66.7 | Station ICE1 JR291 (-60.21 °N, -46.34 °E) |
|  |  | 165 |  | 75.5 |  |
|  |  | 76 | December | 33.0 | Station ICE2 JR291 (-59.96 °N, -46.16 °E) |
|  |  | 178 |  | 154.1 |  |
|  |  | 61 | November | 68.0 | Station ICE2 JR304 (-59.96 °N, -46.16 °E) |
|  |  | 163 |  | 77.3 |  |
| Cadée et al. (1992)^5^ | Scotia-Weddell seas * | 50 | December | *166* | Smaller size FP at 50 m |
|  |  | 150 |  | *45* |  |
|  |  | 75 | December | *220* | Krill swarm observed |
|  |  | 150 |  | *205* |  |
| Wefer at al. (1988)^6^ ^b^ | Bransfield Strait | 494 | January | 281.2 | Productive period, krill FP dominant |
|  |  | 1588 |  | 139.9 |  |
| Accornero et al. (2003)^7^ | Ross  sea * | 180 | Annual mean | 0.05 | Cylindrical faecal fragments – believed to be krill or large copepods |
|  |  | 868 |  | 0.03 |  |
| Cavan et al. (2015)^8^ ^c^ | Scotia Sea * | 70 | January | 58.6 | Marginal ice zone station 12, krill FP dominant |
|  |  | 170 |  | 77.9 |  |
| González (1992)^9^ ^d^ | Scotia-Weddell seas * | 50 | December-January | 10 | Transect (I) (-57 to -62 °N at -49 °E) |
|  |  | 150 |  | 5 |  |
|  |  | 50 | December-January | 5.5 | Transect (II) (-57 to -62 °N at -47 °E) |
|  |  | 150 |  | 0.5 |  |
|  |  | 50 | December-January | 3 | Transect (III) (-57 to -62 °N at 49 °E) |
|  |  | 150 |  | 10.5 |  |
|  |  | 300 |  | 9 |  |
|  |  | 50 | December-January | 22.5 | Station 157 (-59 °N, 49 °E) |
|  |  | 150 |  | 1.5 |  |

^a^ All fluxes refer to the carbon flux, with the exception of Cadée et al. (1992)^5^ where fluxes are given in terms of the number of krill FP strings

* Observations made in the MIZ are marked with a star

^b^ Fluxes are for total particulate organic carbon

^c^ Fluxes are for all FP, but krill FP were dominant

^d^ Fluxes are FP in terms of FP dry weight, and have been estimated from Fig. 3, Fig. 5 of González (1992)^9^

**Supplementary References**

1. Henson, S. A. *et al.* A reduced estimate of the strength of the ocean’s biological carbon pump. *Geophys. Res. Lett.* **38,** L04606 (2011).

2. Dunne, J. P., Sarmiento, J. L. & Gnanadesikan, A. A synthesis of global particle export from the surface ocean and cycling through the ocean interior and on the seafloor. *Global Biogeochem. Cycles* **21,** (2007).

3. Laws, E. A., Falkowski, P. G., Smith, W. O., Hugh, D. & Mccarthy, J. J. Temperature effects on export production in the open ocean. *Global Biogeochem. Cycles* **14,** 1231–1246 (2000).

4. Belcher, A. *et al.* The potential role of Antarctic krill faecal pellets in efficient carbon export at the marginal ice zone of the South Orkney Islands in spring. *Polar Biol.* **40,** (2017).

5. Cadée, G. C., González, H. E. & Schnack-Schiel, S. B. Krill diet affects faecal string settling. *Polar Biol.* **12,** 75–80 (1992).

6. Wefer, G., Fischer, G., Fuetterer, D. & Gersonde, R. Seasonal particle flux in the Bransfieid Strait, Antarctica. *Deep Sea Res.* **35,** 891–898 (1988).

7. Accornero, A., Manno, C., Esposito, F. & Gambi, M. C. The vertical flux of particulate matter in the polynya of Terra Nova Bay . Part II . Biological components. *Antarct. Sci.* **15,** 175–188 (2003).

8. Cavan, E. L. *et al.* Attenuation of particulate organic carbon flux in the Scotia Sea, Southern Ocean, controlled by zooplankton fecal pellets. *Geophys. Res. Lett.* **42,** 821–830 (2015).

9. González, H. E. The distribution and abundance of krill faecal material and oval pellets in the Scotia and Weddell Seas (Antarctica) and their role in particle flux. *Polar Biol.* **12,** 81–91 (1992).
